# Optimizing Esophageal Cancer Diagnosis with Computer-Aided Detection by YOLO Models Combined with Hyperspectral Imaging

**DOI:** 10.3390/diagnostics15131686

**Published:** 2025-07-02

**Authors:** Wei-Chun Weng, Chien-Wei Huang, Chang-Chao Su, Arvind Mukundan, Riya Karmakar, Tsung-Hsien Chen, Amey Rajesh Avhad, Chu-Kuang Chou, Hsiang-Chen Wang

**Affiliations:** 1 Department of Gastroenterology, Kaohsiung Armed Forces General Hospital, 2, Zhongzheng 1st. Rd., Lingya District, Kaohsiung City 80284, Taiwan; mikeman7309@gmail.com (W.-C.W.); forevershiningfy@yahoo.com.tw (C.-W.H.); 2 Department of Nursing, Tajen University, 20, Weixin Rd., Yanpu Township, Pingtung County 90741, Taiwan; 3 Division of Gastroenterology and Hepatology, Department of Internal Medicine, Ditmanson Medical Foundation Chia-Yi Christian Hospital, Chiayi 60002, Taiwan; 06155@cych.org.tw; 4 Department of Mechanical Engineering, National Chung Cheng University, 168, University Rd., Min Hsiung, Chiayi 62102, Taiwan; d09420003@ccu.edu.tw (A.M.); karmakarriya345@gmail.com (R.K.); 5 Department of Internal Medicine, Ditmanson Medical Foundation Chiayi Christian Hospital, Chiayi 60002, Taiwan; cych13794@gmail.com; 6 Department of Computer Science, Sanjivani College of Engineering, Station Rd., Singapur, Kopargaon 423603, Maharashtra, India; ameyavhadcomp@sanjivanicoe.org.in; 7 Obesity Center, Ditmanson Medical Foundation Chia-Yi Christian Hospital, Chiayi 60002, Taiwan; 8 Department of Medical Research, Buddhist Tzu Chi Medical Foundation, Dalin Tzu Chi Hospital, No. 2, Minsheng Road, Dalin, Chiayi 62247, Taiwan; 9 Hitspectra Intelligent Technology Co., Ltd., Kaohsiung 80661, Taiwan

**Keywords:** esophageal cancer, hyperspectral imaging, SAVE, dysplasia, SCC YOLOv5, YOLOv8, narrow-band imaging, white-light imaging

## Abstract

**Objective**: Esophageal cancer (EC) is difficult to visually identify, rendering early detection crucial to avert the advancement and decline of the patient’s health. **Methodology**: This work aimed to acquire spectral information from EC images via Spectrum-Aided Visual Enhancer (SAVE) technology, which improves imaging beyond the limitations of conventional White-Light Imaging (WLI). The hyperspectral data acquired using SAVE were examined utilizing sophisticated deep learning methodologies, incorporating models such as YOLOv8, YOLOv7, YOLOv6, YOLOv5, Scaled YOLOv4, and YOLOv3. The models were assessed to create a reliable detection framework for accurately identifying the stage and location of malignant lesions. **Results**: The comparative examination of these models demonstrated that the SAVE method regularly surpassed WLI for specificity, sensitivity, and overall diagnostic efficacy. Significantly, SAVE improved precision and F1 scores for the majority of the models, which are essential measures for enhancing patient care and customizing effective medicines. Among the evaluated models, YOLOv8 showed exceptional performance. YOLOv8 demonstrated increased sensitivity to squamous cell carcinomas (SCCs), but YOLOv5 provided reliable outcomes across many situations, underscoring its adaptability. **Conclusions**: These findings highlight the clinical importance of combining SAVE technology with deep learning models for esophageal cancer screening. The enhanced diagnostic accuracy provided by SAVE, especially when integrated with CAD models, offers potential for improving early detection, precise diagnosis, and tailored treatment approaches in clinically pertinent scenarios.

## 1. Introduction

Esophageal cancer (EC) has a 5-year survival rate of 15 to 20%, with a lifetime risk of developing this cancer being 0.8% for men and 0.3% for women, which increases with age [1]. EC can be broadly categorized into two types: esophageal squamous cell carcinoma (ESCC) and Esophageal Adenocarcinoma (EAC) [2]. EC is relatively uncommon in the United States; although around 18,440 people were diagnosed and over 16,170 died from it in 2020, it ranks only as the 11th most common cause of cancer death in the country [3]. Early detection of EC is vital for increasing survival rates and lowering morbidity and lethality. Achalasia is a distinct risk factor for esophageal squamous cell carcinoma, as prolonged stagnation and mucosal lesions ultimately induce neoplastic changes. Consequently, the choice of treatment influences the degree of symptom relief and complication rates and may also have secondary implications for long-term cancer risks. For instance, more permanent myotomy techniques (laparoscopic Heller or POEM) exhibit a superior impact on reducing the severity of symptoms compared to pneumatic dilation or botulinum toxin, potentially resulting in a diminished incidence of dysplastic transformations. The data regarding treatment modalities and early detection efforts for esophageal neoplasia indicate the necessity to optimize functional outcomes while also employing sensitive imaging techniques, such as SAVE, to monitor this high-risk population.

ESCC usually starts from the squamous epithelial lining of the esophagus and develops in the middle or upper section of the esophagus. Conversely, EAC typically arises from glandular cells close to the stomach and occurs in the lower middle portion of the esophagus [4]. Globally, ESCC remains the dominant histologic subtype, particularly in East Asia and parts of Africa. Although the incidence of EAC has been rising in several high-income countries, including the United States, Australia, and Western Europe, ESCC still comprises the majority of cases in North America. EAC generally carries a somewhat better prognosis than ESCC when detected at an early stage [5,6]. EAC generally has higher median survival rates compared to ESCC, especially in cases diagnosed at an early stage [7]. Dysplasia refers to abnormal tissue development that indicates neoplasia or a pre-neoplastic state, potentially leading to cancer [8,9]. According to current theories, EAC develops through a sequence of histological phases, which begin with non-dysplastic Barrett’s esophagus (BE), followed by low-grade dysplasia (LGD), then high-grade dysplasia (HGD), and finally result in EAC [10]. When HGD occurs, the risk of developing EC can be greater than 10% per patient-year [11].

Recent advancements in medical imaging have revolutionized EC detection and classification, offering a new level of precision and accuracy. Chen et al. [12] developed an improved Faster RCNN for esophageal cancer detection (ECD) using 1520 gastrointestinal CT images from 421 patients. They achieved 92.15% mAP and an F-1 measure of 95.71%. de Groof et al. [13] developed a hybrid ResNet-UNet-based CAD system for classifying images such as neoplasms or nondysplastic Barret’s esophagus (BE), the pretraining was performed using 494,364 labelled endoscopic images and was tested using dataset 4 consisting of 80 images and received an accuracy of 89% and a 90% sensitivity. Cai et al. [14] developed a CNN-based system and worked with a dataset consisting of 2428 esophagoscopy images, which contained 1332 abnormal and 1096 standard images, and a validation set of 187 images, achieving an accuracy of 91.4% and a specificity of 97.8%. Ghatwary et al. [15] evaluated multiple CNN-based object detection methods to identify EAC regions from high-definition white-light endoscopy (HD-WLE) images. The SSD model achieved the best results with an average recall rate of 0.81, average precision rate of 0.69, and sensitivity of 0.93, based on data from 39 patients. Wu et al. [16] proposed a DCNN (Deep CNN)-based system for automatic esophageal lesion classification and segmentation. It employed Faster-RCNN as the localization module and a Dual-Stream Network as the classification module and achieved an accuracy of 96.28% when tested using 1051 white-light esophageal images. These technologies are not just advanced but reliable and accurate, providing reassurance to both medical professionals and patients.

In addition to these advancements, technologies such as hyperspectral imaging (HSI) and narrow-band imaging (NBI) are also transforming diagnostic capabilities in imaging. Hyperspectral imaging is a technique that creates a 3D hypercube with spatial and spectral dimensions. In this method, pixel values are captured across numerous narrow wavelength bands to produce unique material spectral signatures that offer high spectral resolution and continuous profiles, better identifying and characterizing objects than traditional RGB imaging [17]. HSI captures comprehensive spectral data by sampling the reflective range of the electromagnetic spectrum from 400 to 2400 nanometers, utilizing hundreds of closely spaced spectral bands [18]. It contains many bands ranging from hundreds to thousands, each organized within a narrower bandwidth of 5 to 20 nm [19]. HSI has applications in various fields like the military [20], food industry [21], pharmaceutical industry [22], identification of gaseous and solid objects [23], etc. In art conservation, it is used to detect damage and previous interventions by comparing different spectral bands instead of the orthodox method of UV-fluorescence imaging [24]. Moreover, HSI also allows for an accurate, time-and-cost-efficient mapping of the classification of landscape-scale vegetation from a remote area compared to conventional methods [25].

Narrow-band imaging (NBI) is an innovative technique that improves the diagnostic capabilities of endoscopes in tissue characterization by utilizing narrow-bandwidth filters within an RGB sequential illumination system [26]. NBI uses narrow-band illumination with center wavelengths of 415 nm (blue channel) and 540 nm (red channel), as it matches with the absorption peak of hemoglobin to enhance the contrast of capillary images, which are challenging to observe with traditional White-Light Imaging (WLI) [27]. Barrueto et al. [28] conducted a study where patients were imaged with WLI and NBI. NBI detected four lesions missed by white light, and among 255 confirmed endometriosis lesions, NBI identified all of them with 100% sensitivity, while WLI detected them with 79% sensitivity. The study conducted by Ezoe et al. [29] also demonstrated that NBI provided a more precise identification of gastric, small depressive lesions (SDLs) compared to WLI, with an accuracy of 79% vs. 44% and a sensitivity of 70% vs. 33%.

In this research, we transformed WLI images into SAVE images using the SAVE imaging technique. We trained several object detection models—including YOLOv8, YOLOv7, YOLOv6, YOLOv5, Scaled YOLOv4, and YOLOv3—on both the WLI and SAVE datasets to evaluate the efficacy of SAVE images in early esophageal cancer detection. The results consistently demonstrated that across all models, the SAVE dataset outperformed WLI in identifying early-stage esophageal cancer. This suggests that the SAVE imaging technique could be highly effective in medical institutions for improving early diagnosis and patient outcomes in esophageal cancer.

## 2. Materials and Methods

### 2.1. Dataset

**Figure 1:**
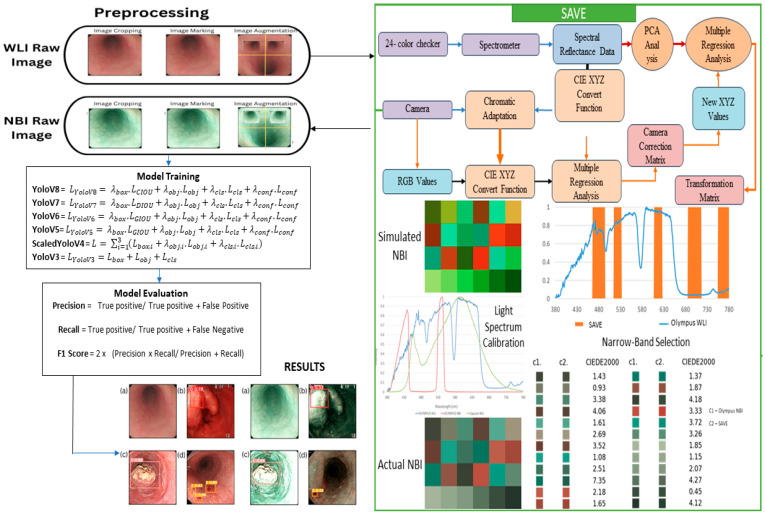
Overall flowchart of the study.

The dataset used in this study was acquired from Kaohsiung Medical University utilizing a traditional endoscope, specifically the CV-290 Olympus model. Initially, the dataset comprised 2063 images, all captured at Kaohsiung Medical University. This dataset was categorized into 11 distinct classes: dysplasia, SCC, bleeding, inflammation, throat, cardia, objects, tools, artificial information, bubbles, and reflection. Given the focus on the early detection of esophageal cancer (EC), particular emphasis was placed on the detection of dysplasia, which is recognized as a precursor to SCC. To enhance the robustness and diversity of the dataset, various data augmentation techniques were applied. These techniques included horizontal flips, vertical flips, and random rotations, which rotated random images by 90 degrees, which helped increase the overall image count to 4125. Specifically, the number of dysplasia images increased from 601 to 1202, and the SCC images increased from 159 to 318 (see Table S1 for the dataset details before and after augmentation). The SAVE dataset was generated by applying the SAVE technique to the WLI images. All the photos were cropped to or had a maximum pixel size of 640 × 640 while importing the dataset in the models. Both datasets were then systematically divided into training, validation, and testing subsets, following a 7:2:1 split, respectively. This careful partitioning ensured a comprehensive evaluation of the model’s performance across different phases of training and testing. To mitigate the severity of class imbalance, a class-weighted focal loss was employed, where per-class weights were inversely proportional to the square root of the sample count for each class, thereby preferentially down-weighting more prevalent classes and up-weighting rarer classes. A stratified batch sampling method that guaranteed each class had at least one alternate instance per mini-batch was utilized, occurring consistently throughout training, thereby mitigating the overrepresentation of the minority in parameter updates. In future endeavors, we will implement class-stratified partitioning—specifically for underrepresented SCC and precancerous categories—to enhance the stability of multi-class training. The overall flow of the project is shown in Figure 1.

### 2.2. SAVE (Spectrum-Aided Vision Enhancer)

The SAVE approach dramatically inspired imaging research and allowed researchers to conceive new color science and imaging technology. In creating the SAVE dataset for this research, WLI photos were converted into HSI images. Before converting a WLI image into other colors (NBI), the image had to be calibrated with a spectrometer. The process began with the calibration of the endoscope’s camera using the Macbeth Color Checker, a widely recognized standard for color accuracy. The Macbeth Color Checker, also known as the X-Rite Classic, contains 24 colored squares representing a diverse range of hues commonly encountered in natural settings, such as red, green, blue, cyan, magenta, and yellow, along with six shades of gray. In this research, X-Rite was the tool of preference for color calibration as it agrees with the colors which were produced by the endoscopic camera. The 24-color patch image during transformation was converted to the CIE 1931 XYZ color space. Calibration was conducted utilizing an Olympus CV-290 endoscopic camera in conjunction with its integrated LED white-light source within a dimly lit environment to exclude ambient light. The Macbeth Color Checker was positioned perpendicular to the optical axis at a constant distance of 2 cm from the distal tip. The camera settings were fixed at an aperture of f/2.8, a shutter speed of 1/25 s, and a gain equivalent to ISO 400, with both auto-exposure and auto-white-balance turned off. A one-point manual white balance was conducted on the 18% gray patch prior to each session, and no further post-capture illumination adjustments were made.

Once the endoscope had captured an image of the Macbeth Color Checker, the image was stored in the standard RGB (sRGB) color space. The sRGB values, which range from 0 to 255, were normalized to a range between 0 and 1 to facilitate further processing.

These normalized values were then subjected to a Gamma correction function following Equation (1):

(1) $$f\left(n\right)=\left\{\begin{array}{c} {\left(\frac{n+0.055}{1.055}\right)}^{2.4},\;n>0.04045\\ \left(\frac{n}{12.92}\right),\;otherwise \end{array}\right.$$

These Gamma-corrected values are referred to as linearized RGB values because they more accurately represent the true colors of the scene as perceived by human vision. Next, the linearized RGB values were transformed into the CIE 1931 XYZ color space. The XYZ color space is a device-independent model used to describe colors in a way that aligns with human vision, making it a critical intermediary in color processing. The transformation from sRGB to XYZ was accomplished using a transformation matrix, as shown in Equation (2):

(2) $$\left[\begin{array}{c} X\\ Y\\ Z \end{array}\right]=\left[M_{A}\right]\left[T\right]\left[\begin{array}{c} f\left(R_{sRGB}\right)\\ f\left(G_{sRGB}\right)\\ f\left(B_{sRGB}\right) \end{array}\right]\times 100,\;0\leq \begin{array}{c} R_{sRGB}\\ G_{sRGB}\\ B_{sRGB} \end{array}\leq 1$$

Images captured by an endoscope can suffer from various imperfections such as non-linear responses, dark current, and color distortions. Non-linear responses occur due to the sensor’s behavior, which may not linearly correlate with the intensity of light it receives, leading to inaccuracies in color representation. Dark current refers to the noise generated by the endoscope sensor even in the absence of light, which can skew the color data. Color distortion can arise from various factors, including incorrect color separation by the endoscope’s sensor.

To correct these errors, a correction matrix C was derived based on the comparison between the actual spectrometer-measured XYZ and the calculated XYZ values from the RGB image. The corrected XYZ values XYZ Correct were computed using Equations (3) and (4):

(3) $$\left[C\right]=\left[{\mathrm{XYZ}}_{\mathrm{Spectrum}}\right]\times \mathrm{pinv}\left(\left[V\right]\right)$$

(4) $$\left[{\mathrm{XYZ}}_{\mathrm{Corrcnt}}\right]=\left[C\right]\times \left[V\right]$$

After correcting the XYZ values, the next step involved converting these values into the Lab color space. The Lab color space is designed to be perceptually uniform, meaning that the numerical differences between colors in this space correspond to perceived differences in color. This makes it particularly useful for calculating color differences.

The conversion from XYZ to Lab was carried out using Equations (5) and (6).

(5) $$\begin{array}{c} L^{*}=116f\left(\frac{Y}{Y_{n}}\right)-16\\ \begin{array}{c} a^{*}=500\left[f\left(\frac{X}{X_{n}}\right)-f\left(\frac{Y}{Y_{n}}\right)\right]\\ b^{*}=200\left[f\left(\frac{Y}{Y_{n}}\right)-f\left(\frac{Z}{Z_{n}}\right)\right] \end{array} \end{array}$$

(6) $$f\left(n\right)=\left\{\begin{array}{c} n^{\frac{1}{3}},\;n>0.008856\\ 7.787n+0.137931,\;otherwise \end{array}\right.$$

By converting XYZ to Lab, the algorithm could calculate color differences using the CIEDE 2000 formula, which provides a more accurate measure of perceived color differences. The spectrometer used in the study was the Ocean Optics QE65000, which was compatible with the X-Rite board. The study obtained the reflectance spectrum of the 24-color patch using that spectrometer. The brightness ratio was derived from the Y value of the XYZ color gamut space, as this parameter directly correlates with brightness. The reflectance spectrum data were converted into XYZ values, which were then normalized within the XYZ color gamut space. A correction coefficient matrix was derived through multiple regression analysis, specifically using a defined equation. The reflectance spectrum data were also used to calculate the transformation matrix for the colors included in the X-Rite board.

Principal Component Analysis (PCA) was conducted on the reflectance spectrum dataset to extract the six most significant principal components (PCs) and their corresponding eigenvectors. These six PCs accounted for 99.64% of the information. The mean root-mean-square error (RMSE) of the 24 desired colors between the corrected XYZ values and the spectrally measured XYZ values was 0.19, indicating that the difference was insignificant. Further analysis was conducted to explore the relationship between the transformation matrix and the six significant principal components, leading to the computation of the analog spectrum from the corrected XYZ values. The RMSE of the 24 color blocks was 0.056, and the average color discrepancy between the simulated analog spectrum and the spectrally measured reflectance spectrum was 0.75, suggesting that the colors closely matched the observed values. This demonstrated that the developed technique could effectively convert an RGB image captured by an endoscope into an HSI image.

The transformation matrix M was calculated using the scores obtained from PCA, allowing the simulation of the spectrum SSpectrum from the corrected XYZ values using Equations (7) and (8):

(7) $$\left[M\right]=\left[\mathrm{Score}\right]\times \mathrm{pinv}\left(\left[V_{\mathrm{Color}}\right]\right)$$

(8) $${\left[\mathrm{SSpectrum}\right]}_{380\sim 780\mathrm{nm}}=\left[\mathrm{EV}\right]\left[M\right]\left[V_{\mathrm{Color}}\right]$$

The root-mean square error (RMSE) was then calculated between the simulated and observed XYZ values for each of the 24 color squares on the Macbeth Color Checker. This error metric provided a quantitative measure of how closely the simulated colors matched the actual colors. The matrix C correction coefficient was used for a regression of the endoscope errors on V. From the data for XYZ_Correct_ vs. XYZ_Spectrum_, the average RMSE was 0.5355.

The HSI conversion method was developed to detect and classify EC and WLI images to SAVE with the assistance of Olympus. An actual SAVE image was obtained by the Olympus endoscope; the simulated SAVE image and actual SAVE image were compared with the average 24-color Macbeth checker. The CIEDE 2000 color differences between 24 color blocks were determined and minimized to find the average color difference of 2.79. Three reasons may be cited to justify the difference in color between real and modelled SAVE images: spectrum of light; color matching function; and spectrum of reflection. The light spectrum was normalized by a Cauchy–Lorentz distribution using Equation (9):

(9) $$f\left(x;x_{0},\gamma \right)=\frac{1}{\pi \gamma \left[1+{\left(\frac{x-x_{0}}{\gamma }\right)}^{2}\right]}=\frac{1}{\pi }\left[\frac{\gamma }{{\left(x-x_{0}\right)}^{2}+\gamma^{2}}\right]$$

SAVE and real Olympus NBI image were again normalized with Macbeth 24-colour checkers. It has been noticed that the peak absorption wavelengths of hemoglobin lie between 415 to 540 nm. However, the real NBI picture from the Olympus endoscope had brown in it besides the green and the blue that corresponded to the wavelength of 650 nm. Because of this, it would be appropriate to mention that the NBI videos were subjected to a certain amount of post-processing to obtain a better impression of realism. As such, the study included a light spectrum at 415 and 540 nm in three more locations, i.e., in the wavelength range of 600, 700, and 780 nm.

### 2.3. ML Algorithms

#### 2.3.1. Yolo V8

YOLOv8 is the next generation in the YOLO line of development to make a big stride in throughput, flexibility, and ease of use in real-time object detection. It is built from the strength of its predecessors, amalgamating several key innovations in architecture. It makes use of a CSP-Darknet53 backbone, refined from Darknet53 and augmented by Cross Stage Partial Networks (CSPs). CSPNet further facilitates the flow of gradients and reduces computation complexity; thus, it makes the model more effective and scalable. In the network neck, there is a PANet, which effectively captures features across diverse scales. The bounding-box regression using a CIoU loss involves the inclusion of an overlap area, the distance between the bounding boxes, and aspect ratio differences for more robustness in estimating positions of the bounding box. The equation for the CIoU loss function is shown in Equation (10).

(10) $$CIoU=1-IoU+\frac{\rho^{2}\left(b,b^{g}\right)}{c^{2}}+\alpha v$$

where $\rho \left(b,b^{g}\right)$ is the Euclidean distance between the predicted and ground-truth box centers, c is the length of the diagonal of the smallest enclosing box covering the two boxes, and αv accounts for the aspect ratio consistency. Objectness predicts the presence of an object in each cell of a certain feature map using Equation (11):

(11) $$Objectness\;Loss=-\left[ylog\left(p\right)+\left(1-y\right)log\left(1-p\right)\right]$$

where y is the ground-truth label (1 if the object is present, 0 otherwise) and p is the predicted probability. The classes of objects can either be detected by YOLOv8 using cross-entropy loss or focal loss, wherein the latter helps deal with class imbalance by putting more emphasis on hard-to-classify examples. The math behind focal loss is shown in Equation (12):

(12) $$Focal\;Loss=-\alpha_{t}{\left(1-p_{t}\right)}^{\gamma }\log\left(p_{t}\right)$$

where $p_{t}$ denotes the probability to predict the true class, $\alpha_{t}$ is a balancing factor, and γ is the focusing parameter that provides down-weighting for easy examples. The loss function for YOLOV8 is defined in Equation (13):

(13) $$L_{YoloV8}=\lambda_{box}\cdot L_{CIOU}+\lambda_{obj}\cdot L_{obj}+\lambda_{cls}\cdot L_{cls}+\lambda_{conf}\cdot L_{conf}$$

#### 2.3.2. Yolo V7

The YOLOv7 model represents the evolution of the YOLO series designed to further advance the frontiers of real-time object detection by offering better accuracy, speed, and efficiency. Core to YOLOv7 is a custom backbone network that balances speed with accuracy. It integrates an Extended Efficient Layer Aggregation Network into the backbone, extending the layer aggregations and thereby empowering the model capability of learning deep features. The architecture also follows a modified version of the CSPNet-Cross Stage Partial Network, which reduces further computational complexity with high accuracy, hence making YOLOv7 more scalable and versatile for various use cases. The key updates in YOLOv7 deal with an enhanced neck consisting of a PANet along with additional SPP modules. The SPP modules are responsible for reliable feature extraction by pooling features at different scales, hence promoting multi-resolution object processing.

YOLOv7 features advanced loss functions in order to optimize detection performance. The bounding-box regressions rely on a loss function called Distance-IoU (DIoU) that considers the overlap area along with the distance between the predicted and ground-truth bounding boxes to provide more accurate localization, as shown in Equation (14):

(14) $$DIoU=1-IoU+\frac{\rho^{2}\left(b,b^{gt}\right)}{c^{2}}$$

where $\rho \left(b,b^{gt}\right)$ is the Euclidean distance between the centers of the predicted and ground-truth boxes, and c is the diagonal length of the smallest enclosing box. This method has less risk of poor localization, especially for objects that are close but not perfectly overlapping.

Objectness loss in YOLOv7 is taken care of by a binary cross-entropy loss similar to previous versions, so as to provide correct object presence prediction inside the feature maps as shown in Equation (15):

(15) $$Objectness\;Loss=-\left[ylog\left(p\right)+\left(1-y\right)log\left(1-p\right)\right]$$

where y is the true label, where 1 means that there is an object and 0 otherwise, and p is the predicted probability. YOLOv7 either uses cross-entropy loss or focal loss for the object classification task. For class imbalance, it is more useful to use focal loss, as it focuses on these examples that are hard to classify; it is defined in Equation (16):

(16) $$Focal\;Loss=-\alpha_{t}{\left(1-p_{t}\right)}^{\gamma }\log\left(p_{t}\right)$$

where $p_{t}$ is the predicted probability of the true class, $\alpha_{t}$ is a balancing factor, and γ is the focusing parameter that reduces the weight of easy examples. The loss function for YOLO7 is defined in Equation (17).

(17) $$L_{YoloV7}=\lambda_{box}\cdot L_{DIOU}+\lambda_{obj}\cdot L_{obj}+\lambda_{cls}\cdot L_{cls}+\lambda_{conf}\cdot L_{conf}$$

#### 2.3.3. YOLOV6

Compared with previous models of the YOLO series, YOLOv6 is a big step forward in which boundaries are pushed both in speed and accuracy. In contrast, the backbone of YOLOv6 features an improved CSPNet with an intrinsic design to enhance the feature extraction process by incorporating Cross Stage Partial (CSP) networks. This backbone is also referred to as the CSPRepResNet backbone and leverages the ResNet backbone architecture by combining the advantages of residual connections and the extension of the gradient flow given by CSPNet. It further improves efficiency with RepVGG-style blocks in the backbone, which allows the model to enjoy a faster inference speed while not losing accuracy. The neck in YOLOv6 is based on the PANet architecture, with a revised design to allow for freer flow of information at multiple layers and scales.

YOLOv6 uses a combination of loss functions to effectively optimize the object detection process. It uses the GIoU loss for bounding-box regression, which is an extension from the standard IoU. It considers not only the overlapping area between the predicted and ground-truth bounding boxes but also the consistency in distance and size. The GIoU loss is defined in Equation (18):

(18) $$GIoU=IoU-\frac{A_{c}-\left(A_{u}+A_{i}\right)}{A_{c}}$$

where $A_{c}$ is the area of the smallest enclosing box which covers both the predicted and ground-truth boxes, $A_{u}$ is a union area of these boxes, and $A_{i}$ is an intersect area. This loss function further strengthens the condition of robustness and speeds up the convergence by providing a more comprehensive measure of how well the predicted box and the ground-truth box are aligned.

Finally, YOLOv6 uses binary cross-entropy loss for objectness prediction as shown in Equation (19):

(19) $$Objectness\;Loss=-\left[ylog\left(p\right)+\left(1-y\right)log\left(1-p\right)\right]$$

where y is a ground-truth label (1 if the object is present, 0 otherwise), and p is a predicted probability. This loss function is able to appropriately guide the network to clearly discriminate between object and background regions. In classification, YOLOv6 applies focal loss when addressing class imbalance, so that it focuses more on hard-to-classify examples, as shown in Equation (20):

(20) $$Focal\;Loss=-\alpha_{t}{\left(1-p_{t}\right)}^{\gamma }\log\left(p_{t}\right)$$

where $p_{t}$ is a predicted probability for the true class, $\alpha_{t}$ is a balancing factor, and γ is a focusing parameter that down-weights easy examples and up-weights the harder, misclassified examples during training. The loss function for YOLOV6 is displayed in Equation (21):

(21) $$L_{YoloV6}=\lambda_{box}\cdot L_{GIOU}+\lambda_{obj}\cdot L_{obj}+\lambda_{cls}\cdot L_{cls}+\lambda_{conf}\cdot L_{conf}$$

#### 2.3.4. YoloV5

YOLOv5 is the fifth model in the YOLO series, which improves over its predecessors in terms of speed, accuracy, and ease of use for real-time object detection. The backbone of YOLOv5 is a CSPNet-enhanced version of the original Darknet. It contains CSP networks that enhance the model’s learning ability. The CSPNet enhances the learning capacity of the model through two main integral parts: the feature map of the base layer and its cross-stage hierarchy. The head of YOLOv5 consists of the Path Aggregation Network (PANet) for better feature map fusion from various layers, which makes multi-scale object detection better and more accurate.

YOLOv5 uses a combination of loss functions to optimize training. The bounding-box regression loss implements the Generalized Intersection over Union (GIoU) loss. This extends the basic Intersection over Union (IoU) since it does not only consider the area of overlap between the bounding boxes but also their distance and sizes and both ground-truth and predicted ones. The GIoU loss is defined in Equation (22):

(22) $$GIoU=IoU-\frac{A_{c}-\left(A_{u}+A_{i}\right)}{A_{c}}$$

where $A_{c}$ is the area of the smallest enclosing box of the predicted and ground-truth boxes, $A_{u}$ is the union area for both boxes, and $A_{i}$ is the area of intersection. This loss function increases the robustness and convergence speed for cases where the predicted boxes are located quite a distance from the ground truth. Moreover, for both objectness and classification, a combination of binary cross-entropy loss functions is used by YOLOv5. The loss definition of binary cross-entropy as defined in Equation (23):

(23) $$Objectness\;Loss=-\left[ylog\left(p\right)+\left(1-y\right)log\left(1-p\right)\right]$$

where y is the ground-truth label (i.e., an object is either present or absent), and p is the predicted probability; it then goes on to the next step. The obtained classification output scores for all classes are further operated to apply a softmax function, followed by the cross-entropy loss for computing classification accuracy. The loss function for YOLOV5 is defined in Equation (24):

(24) $$L_{YoloV5}=\lambda_{box}\cdot L_{GIOU}+\lambda_{obj}\cdot L_{obj}+\lambda_{cls}\cdot L_{cls}+\lambda_{conf}\cdot L_{conf}$$

#### 2.3.5. ScaledYoloV4

Scaled-YOLOv4 is a scaled version of the YOLOv4 series for a variety of model sizes while maintaining performance in real-time object detection. Compared with the original YOLOv4 baseline, Scaled-YOLOv4 introduces several new architectural enhancements, optimized training strategies, and scaling methods that substantially improve both the efficiency and accuracy of the model, hence being very effective in a wide variety of applications concerning autonomous driving, surveillance, and medical imaging.

Scaled-YOLOv4 has the backbone of CSPDarknet53, which is an improved version of Darknet53 with a CSP (Cross Stage Partial) network. CSPDarknet53 divides the feature map into two parts, one of which undergoes additional convolution processing while the other bypasses it, trying to promote better feature fusion to reduce redundant gradient information. In Scaled-YOLOv4, the neck incorporates a Path Aggregation Network PANet and a Spatial Pyramid Pooling SPP module for feature aggregation. PANet reinforces the information transmission between layers, encapsulating richer semantic information at more scales, which is very important for the detection of objects of various sizes in complicated scenarios. Via further refinement, SPP pools the features of various scales and then concatenates them to allow the model to retain spatial information and therefore enhances the detection of small objects.

Scaled-YOLOv4 adopts a variety of loss functions to tackle different aspects of the object detection task. Concerning the bounding-box regression, the Complete Intersection over Union loss is used, which further extends the IoU loss with more metrics such as including the overlap area, the distance between the predicted and ground-truth box centers, and the consistency of the aspect ratio. The CIoU loss is defined in Equation (25):

(25) $$CIoU=1-IoU+\frac{\rho^{2}\left(b,b^{g}\right)}{c^{2}}+\alpha v$$

where $\rho \left(b,b^{g}\right)$ is the Euclidean distance between the predicted and ground-truth box centers, c is the diagonal length of the smallest enclosing box covering both the predicted and ground-truth boxes, and αv is the aspect-ratio consistency term. By adding this loss function, robustness and convergence may be enhanced, especially when bounding-box alignment is not so good.

Scaled-YOLOv4 uses binary cross-entropy loss for objectness prediction as shown in Equation (26):

(26) $$ObjectnessLoss=-\left[ylog\left(p\right)+\left(1-y\right)log\left(1-p\right)\right]$$

The terms y indicates the presence, 1, or absence, 0, of an object, and *p* stands for the predicted probability of the presence of any object. It is crucial to train a model on the differences between an object and background regions of an image. These heads make use of a combination of cross-entropy loss and focal loss to handle any class imbalance. Focal loss is defined as

(27) $$FocalLoss=-\alpha_{t}{\left(1-p_{t}\right)}^{\gamma }\log\left(p_{t}\right)$$

where $p_{t}$ is the predicted probability of the true class, $\alpha_{t}$ is a balancing factor, and γ is a focusing parameter that reduces the impact of easily classified examples, which is particularly effective in datasets with a significant class imbalance. The loss function for ScaledYOLOV4 is as follows:

(28) $$L=\sum_{i=1}^{3}\left(L_{box,i}+\lambda_{obj,i}\cdot L_{obj,i}+\lambda_{cls,i}\cdot L_{cls,i}\right)$$

#### 2.3.6. YoloV3

YOLOv3 is the third generation in the YOLO series, which stands for You Only Look Once. It represented a considerable stride toward real-time object detection by mingling speed and accuracy performed admirably on a wide range of applications, from autonomous vehicles to video surveillance and medical imaging.

YOLOv3 is based on Darknet-53, an improved version of the original Darknet architecture with several new features. It contains 53 convolutional layers in total, composed of a series of residual blocks with skip connections similar to the ResNet architecture. While the usage of Leaky ReLU as an activation enhances the power of this network, YOLOv3 works better for detecting objects of different shapes and sizes and also those oriented at various angles. YOLOv3 follows the method of multi-scale detection, which is reinforced by a Feature Pyramid Network (FPN).

YOLOv3 also incorporates several loss functions for its betterment during training. The bounding-box regression loss is based on the mean squared error and calculates the error between the predicted and ground-truth coordinates of the bounding boxes. Then, objectness is determined through binary cross-entropy loss as defined in Equation (29):

(29) $$ObjectnessLoss=-\left[ylog\left(p\right)+\left(1-y\right)log\left(1-p\right)\right]$$

where y denotes the ground-truth class (1 if an object is present and 0 otherwise), and p denotes the predicted probability for an object to be present. This loss penalizes incorrect predictions of object presence or absence, helping the model with a greater difference between object and background regions in the image. Another point concerning classification is that YOLOv3 uses binary cross-entropy loss, instead of the more traditional softmax and cross-entropy combination, as was the case for objectness prediction. The idea is to allow for multi-label classification—the case in which an object might belong to several classes. The loss function for YOLOV3 is shown in Equation (30):

(30) $$L_{YoloV3}=L_{box}+L_{obj}+L_{cls}$$

## 3. Results

**Figure 2:**
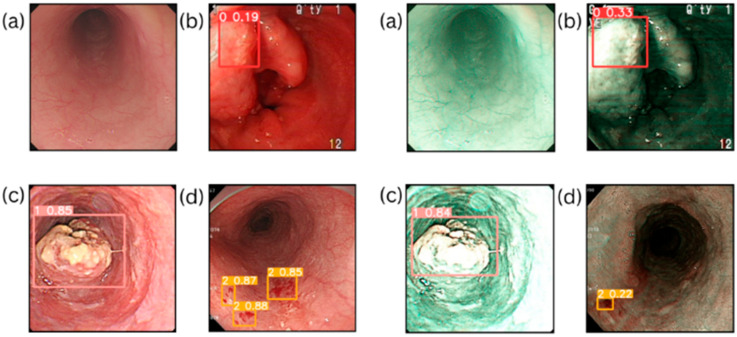
Examples of the diagnostic outcomes for WLI and NBI images of esophageal neoplasms. **Left**: (**a**–**d**) Normal, dysplasia, SCC, and bleeding classes in the WLI imaging modality. **Right**: (**a**–**d**) Normal, dysplasia, SCC, and bleeding classes in the SAVE imaging modality.

The object identification models YOLOv8, YOLOv7, YOLOv5, Scaled YOLOv4, and YOLOv3 were trained for identifying esophageal cancer lesions in this paper using two approaches: WLI and SAVE imaging as shown in Table 1 (see Table S2 for the average metric changes when switching from WLI to SAVE for each YOLO variant). For a full picture, precision and recall measures were used to determine the F1-score and mAP50. All hyperparameters were tuned over 600 epochs of training. Figure 2 also clearly shows that SAVE generally improved the performance of these models, especially in identifying dysplasia and SCC. YOLOv8 resulted in an increase in accuracy as high as 82.1% for dysplasia, after the adaptation of the SAVE technique, compared with 80.9% obtained with WLI. SAVE significantly improved the dysplastic lesions’ recall to 79.5%, compared with the recall achieved by WLI of 70.4%, showing that YOLOv8 had an improved capability for accurate detection of dysplastic lesions. SAVE also improved the dysplasia’s F1-score from an overall 75.2% to 80.8%. The notable rise in recall to 81.3% in the SCC case detection, coupled with SAVE, indicates increased sensitivity for the detection of malignant lesions. The effect of the SAVE technique on the F1-score of SCC detection was an improvement from 83.7% to 87.0%. Similarly, in the case of YOLOv8, our experiments indicated remarkable improvements, with mAP50 as high as 84.1% for dysplasia and 86.7% for SCC, from the original 68.3% and 76.5%.

YOLOv3 showed fairly good results, especially for identifying SCC (Figure S1 contains the confusion matrices obtained by training and testing the YoloV3 model, where Figure S1a is the confusion matrix obtained by running the YoloV3 model on the WLI dataset, while Figure S1b is the confusion matrix obtained by running the YoloV3 model on the SAVE dataset). The recall showed an improvement from 60.0% to 65.9% with SAVE, while the precision when identifying dysplasia rose from 69.5% to 74.7%. This shows better detection capability, hence improved sensitivity. The mAP50 also rose from 71.6% to 75.1%, and the F1-score for SCC rose from 63.9% to 71.3% (Figure S2 contains training results in the form of plots for the YOLOv3 model, where Figure S2a shows the training results obtained by running the YoloV3 model on the WLI dataset, while Figure S2b displays the training results obtained by running the YoloV3 model on the SAVE dataset). The ScaledYOLOv4 model showed interesting recall values of 53.4% for dysplasia and 71.8% for SCC (Figure S3 contains the training results in the form of plots for the ScaledYOLOv4 model, where Figure S3a shows the training results obtained by running the ScaledYOLOv4 model on the WLI dataset, while Figure S3b displays the training results obtained by running the ScaledYOLOv4 model on the SAVE dataset). This may be interpreted as the model having the potential to raise sensitivity for both kinds of lesions. The F1-scores for dysplasia and SCC went as high as 76.7% and 62.9%, respectively. Likewise, similar improvements were obtained for ScaledYOLOv4’s mAP50, with the mAP50 for dysplasia increasing by 7.4%, from 41.2% to 48.6%; the same happened for SCC, where the score rose from 63.3% to 68.8%. It is worth noting that YOLOv7’s detection of dysplasia presented convincing improvements with the SAVE method, achieving 76.4% compared to the 72.0% obtained with WLI. Recall for this diagnosis increased to 50.0% with SAVE and demonstrated that the enhanced accuracy of YOLOv7 in identifying dysplasia was better. The F1-score of SAVE-assessed dysplasia showed an increase from 57.2 to 60.4%. This indicated that YOLOv7 had a recall of about 69.4% for SCC lesion detection, which is promising. In the lesion detection part, the F1-score of SCC detection rose from 74.1% to 76.0% in the case of the SAVE model. For dysplasia and SCC detection using YOLOV6, SAVE showed an improvement in mAP50, with increases from 53.1% to 55.8% and 73.2% to 76.8%, respectively (Figure S4 contains the confusion matrices obtained by training and testing the YOLOv6 model, where Figure S4a is the confusion matrix obtained by running the YOLOv6 model on the WLI dataset, while Figure S4b is the confusion matrix obtained by running the YOLOv6 model on the SAVE dataset). Additionally, SAVE provided higher precision in detecting normal tissues, suggesting that it was more effective in distinguishing between different tissue types (Figure S5 contains the precision curves obtained by training and testing the YOLOv6 model. Where Figure S5a is the precision curve obtained by running the YOLOv6 model on the WLI dataset, while Figure S5b is the precision curve obtained by running the YOLOv6 model on the SAVE dataset. Figure S6 contains the recall curves obtained by training and testing the YOLOv6 model. Where Figure S6a is the recall curve obtained by running the YOLOv6 model on the WLI dataset, while Figure S6b is the recall curve obtained by running the YOLOv6 model on the SAVE dataset. Figure S7 contains the F1 curves obtained by training and testing the YOLOv6 model. Where Figure S7a is the F1 curve obtained by running the YOLOv6 model on the WLI dataset, while Figure S7b is the F1 curve obtained by running the YOLOv6 model on the SAVE dataset. Figure S8 contains the precision-recall curves obtained by training and testing the YOLOv6 model. Where Figure S8a is the precision-recall curve obtained by running the YOLOv6 model on the WLI dataset, while Figure S8b is the precision-recall curve obtained by running the YOLOv6 model on the SAVE dataset). With an accuracy of 85.1%, YOLOv5 showed a significant improvement in precision for SCC diagnostics using SAVE. This proved its efficiency at eliminating false positives and therefore at increasing the accuracy of the detection of malignant lesions. The F1-score for SCC increased from 70.4% to 76.2, therefore showing a better recall and precision ratio. YOLOv5’s mAP50 for SCC increased from 73.6% to 75.1%. The overall trend of the models shows that SAVE continuously improved the recall, particularly in those high-risk lesions of dysplastic and SCC cases. Among all these improvements, the largest recall improvement of 79.5% was obtained by YOLOv8, followed by a very significant increase in ScaledYOLOv4. Using the YOLOv8 and ScaledYOLOv4 models improved the recall to 81.3% and 71.8%, respectively, in the identification of SCC. Among these models, YOLOv5 presented an increase in precision for SCC of 85.1% compared to others, highlighting the high capability of this model to detect true positive information with SAVE. It was further seconded by the remarkable enhancements in recall rates of the YOLOv7 models of 69.4% and 76.4% for SCC and dysplasia cases, respectively (Figure S9 contains training results in the form of plots for the YOLOv7 model, where Figure S9a shows the training results obtained by running the YOLOv7 model on the WLI dataset, while Figure S9b displays the training results obtained by running the YOLOv7 model on the SAVE dataset). To sum up, the YOLOv8 obtained excellent metrics overall and was also effective in recalling information about SOPs regarding different types of cancer (Figure S10 contains the confusion matrices obtained by training and testing the YOLOv7 model. Where Figure S10a is the confusion matrix obtained by running the YOLOv7 model on the WLI dataset, while Figure S10b is the confusion matrix obtained by running the YOLOv7 model on the SAVE dataset. Figure S11 contains the precision curves obtained by training and testing the YOLOv7 model. Where Figure S11a is the precision curve obtained by running the YOLOv7 model on the WLI dataset, while Figure S11b is the precision curve obtained by running the YOLOv7 model on the SAVE dataset. Figure S12 contains the recall curves obtained by training and testing the YOLOv7 model. Where Figure S12a is the recall curve obtained by running the YOLOv7 model on the WLI dataset, while Figure S12b is the recall curve obtained by running the YOLOv7 model on the SAVE dataset. Figure S13 contains the F1 curves obtained by training and testing the YOLOv7 model. Where Figure S13a is the F1 curve obtained by running the YOLOv7 model on the WLI dataset, while Figure S13b is the F1 curve obtained by running the YOLOv7 model on the SAVE dataset. Figure S14 contains the precision-recall curves obtained by training and testing the YOLOv7 model. Where Figure S14a is the precision-recall curve obtained by running the YOLOv7 model on the WLI dataset, while Figure S14b is the precision-recall curve obtained by running the YOLOv7 model on the SAVE dataset). The ability to obtain superior F1-scores and mAP50, particularly when the SAVE imaging protocol was used, was the underlying reason behind its remarkable resilience for detecting both dysplasia and SCC. Based on the consistent enhancements across all metrics used for evaluation, YOLOv8 proved to be the best reliable and efficient model for the detection of esophageal cancer lesions in this work, and hence the best choice for clinical applications, where precision and speed are pertinent. The results are shown in Figure 2.

An enhancement of 2% in sensitivity would result in the identification of two additional lesions at an early stage per 100 patients, which would otherwise remain undetected until a more invasive stage. This increase accumulates and can significantly impact subsequent morbidity and treatment expenses. Although our manuscript underwent internal review by imaging scientists and clinicians, a multicenter study is planned to further validate the extent of the improvement these initial gains represent in real-world patient care. This study will include an error analysis, endpoint definitions by clinicians, and statistical significance testing.

## 4. Discussion

This study was dedicated to evaluating the capability of deep learning models in the early detection of EC using various types of medical image data. Models used in our study showed outstanding accuracy, speed, and flexibility that are crucial for real-time applications in healthcare environments. However, several important limitations must be acknowledged, as those may affect the applicability and value of our findings. The dataset utilized for both validation and training purposes had a small number of annotated photos and may not be representative of the variability in the occurrence of symptoms among a greater population. It is an inherent limitation of the present work that we utilized a dataset that was limited. It may be that the number of annotated photos within the validation and training dataset did not introduce enough variety and complexity of symptoms of esophageal cancer occurring within a wider population. This might be another limitation that further reduces the generalization capability of this model to unfamiliar data, which, by implication, reduces its potential clinical effectiveness. However, most of the data provided belonged to a single ethnic group or geographical region, which reduced the bias in performance across the different versions of the models. Models trained on geographically homogenous data could fail to realize optimum performance when applied to diverse patient populations originating from variable geographical areas or ethnicities. This limitation is a concern, especially when researching esophageal cancer, as demographic factors may play a role in the presentation and course of the disease. Again, within our research study, we only considered two types of esophageal cancer: dysplasia and SCC. Though the aforementioned types are the most common, our research study did not take into consideration many of the less common types, including sarcomatoid carcinoma, and other infrequent types. Removing these other types of cancer limits the generalizability of the detection models and may render them less useful in clinical settings where a wider variety of forms of esophageal cancers must be considered. Future studies should aim to develop models designed to identify all types of esophageal cancers, thus providing a more complete diagnostic tool.

In turn, despite such disadvantages, the key advantages of our study are as follows. First, the models used in this paper enable real-time object detection, which is crucial in a clinical environment in view of the fact that timely decision-making can make a great difference for patient outcomes. Accurate detection of small lesions and discrimination from normal tissue confers an enormous advantage in the early diagnosis of esophageal cancer, when early management is critical to improve prognosis. Several ways that the study can be further developed and expanded on for future research are described in the following.

It is essential that larger, more diverse datasets capturing a wide range of imaging diagnoses and patient characteristics are included, comprising all types of esophageal cancers. This will further ensure the robustness of the model for generalization across various demographics and clinical settings with minimal risk of bias, improving its practical utility. Apart from that, transfer learning methods, which pretrain models on large and diverse datasets and fine-tune them on smaller and more specialized datasets, may ultimately improve the accuracy of detection. By doing so, the model leverages knowledge from a larger dataset and enhances its performance on tasks that call for finer skills. There are a lot of practical issues that have to be resolved for the successful implementation of these models in a hospital setting. In future studies, the augmentation methodology should be enhanced by producing domain-aware spectral perturbations that influence wavelength regions deemed physiologically plausible, and by conditioning on authentic lesion spectra generated through GAN-based tissue hyperspectral synthesis to ensure that the results accurately reflect the characteristics of real tissues. Furthermore, we could create adaptive pipelines that gate parameters of the transformation process to specific lesion types and imaging conditions, by integrating informed models of the reflectance process to realistically simulate variations in illumination and tissue absorption, while avoiding non-clinical artifacts and enhancing the robustness of our model. We refrained from employing formal statistical significance tests (e.g., paired *t*-tests or bootstrapped confidence intervals) due to our dataset being an initial, proof-of-concept collection comprising 159 SCC and 601 dysplasia lesions, each yielding a set of paired WLI + SAVE images, thereby violating the independence assumption requisite for such tests. The hypothesis tests would be untrustworthy in this instance. We assessed reproducibility by re-training the model three times with independent random seeds while maintaining identical validation splits of 20% and test splits of 10%. This resulted in minimal variance in mAP (0.5%) and F1 (1.8%) values, demonstrating consistently superior SAVE gains compared to WLI. We acknowledge the significance of formal inference and will incorporate lesion-level confidence limits and hypothesis testing into our broader multicenter studies.

First, these models should be more extensively validated on larger clinical datasets to ensure that their accuracy and reliability are maintained throughout different patient populations, scanning protocols, and malignancies. The process of validation would then help clinicians to gain confidence in the results and establish the validity of the models with greater certainty. This means that it will be very important to try to cooperate with doctors as much as possible to ease the integration of these models into current workflows. This might be taking the time to develop user-friendly software interfaces with ordinary imaging equipment to ensure that the use of AI technologies will enhance established processes without disrupting them. In addition, ongoing training and feedback with healthcare professionals are needed in order to continuously improve the models and adapt them to real clinical scenarios. Feedback provided by clinicians may be considered to find further opportunities for improvement that would definitely address the needs of end-users. It is important to address regulatory issues and data privacy concerns to ensure patient safety and comply with health legislation.

Overcoming these constraints and benefiting from the advantages of deep learning-based models will finally enable us to reach the stage of materializing the capability of AI-based tools for timely identification and classification of all types of esophageal cancers. These can eventually translate into better patient outcomes resulting from earlier diagnosis and more focused therapy and also into more effective workflows that reduce burdens on health systems. Further medical imaging will incorporate AI-based solutions into routine practice, which has the potential to revolutionize cancer diagnosis and improve the quality of patient care worldwide.

## 5. Conclusions

In conclusion, our study provided compelling evidence that the SAVE imaging technique significantly outperformed traditional WLI in the early detection of esophageal cancer. By employing a range of advanced object detection models, including YOLOv8, YOLOv7, and others, we consistently observed superior performance when utilizing SAVE images. This enhanced detection capability is particularly important in the context of early-stage esophageal cancer, where early and accurate diagnosis is critical for effective treatment and improved patient outcomes. The ability of SAVE imaging to highlight subtle visual differences that may go unnoticed in WLI images underscores its potential as a more reliable diagnostic tool. The promising results from this research suggest that integrating SAVE imaging into clinical practice could significantly enhance early cancer detection, potentially leading to earlier diagnoses, more timely interventions, and improved patient prognoses. Additionally, the success of SAVE in this context opens up new possibilities for its application in other areas of medical imaging, where early detection is equally vital. The results strongly indicate SAVE’s potential for improving early EC detection; however, larger multicenter studies, including fully independent external validation and a comprehensive analysis of false positives and negatives in clinically significant categories, are necessary prior to implementation in standard practice. Continued exploration and refinement of this technique could broaden its impact, offering significant advancements in the early diagnosis and treatment of various medical conditions. As medical institutions seek to improve diagnostic accuracy and patient care, the adoption of SAVE imaging could represent a significant step forward in the fight against cancer and other diseases. Future studies will incorporate multi-reader endoscopic and radiological assessments to benchmark SAVE + YOLO performance against expert clinicians, ensuring that the observed algorithmic gains translate into real-world diagnostic improvements.

## Figures and Tables

**Table 1:** The results of WLI and the SAVE imaging modalities of the for the different YOLO models.

Framework	Model	Classes	Metrics			
			Precision in %	Recall in %	F1-Score in %	mAP50 in %
YOLOV8	WLI	Dysplasia	80.900	70.400	75.200	68.300
		SCC	93.400	75.800	83.700	76.500
		Normal	89.200	77.000	82.600	73.300
	SAVE	Dysplasia	82.100	79.500	80.800	84.100
		SCC	93.600	81.300	87.000	86.700
		Normal	94.000	78.800	85.800	89.600
YoloV7	WLI	Dysplasia	72.000	47.500	57.238	46.500
		SCC	84.400	66.000	74.074	68.600
		Normal	70.289	70.951	70.440	69.761
	SAVE	Dysplasia	76.400	50.000	60.443	53.900
		SCC	84.000	69.400	76.005	72.300
		Normal	72.411	65.371	68.554	65.627
YOLOV5	WLI	Dysplasia	70.200	42.000	52.556	48.200
		SCC	71.200	69.700	70.442	73.600
		Normal	63.213	54.929	58.579	57.324
	SAVE	Dysplasia	66.400	43.400	52.491	44.800
		SCC	85.100	69.000	76.209	75.100
		Normal	60.558	53.012	56.367	55.210
YoloV6	WLI	Dysplasia	72.300	49.400	58.700	53.100
		SCC	88.600	69.200	77.700	73.200
		Normal	63.628	59.818	61.521	60.812
	SAVE	Dysplasia	73.800	49.200	59.100	55.800
		SCC	86.000	68.200	76.100	76.800
		Normal	65.428	55.229	59.618	58.076
ScaledYoloV4	WLI	Dysplasia	74.900	47.500	58.133	41.200
		SCC	91.900	65.400	76.418	63.300
		Normal	65.351	56.178	60.256	49.968
	SAVE	Dysplasia	76.400	53.400	62.862	48.600
		SCC	82.400	71.800	76.736	68.800
		Normal	63.997	57.428	60.448	51.824
YOLOV3	WLI	Dysplasia	69.500	38.800	39.400	49.800
		SCC	88.600	60.000	63.900	71.600
		Normal	56.713	57.065	53.674	56.369
	SAVE	Dysplasia	74.700	45.100	45.800	56.200
		SCC	87.400	65.900	71.300	75.100
		Normal	56.282	52.865	51.158	53.969

## Data Availability

Data are provided within Supplementary Information Files. If the editorial department or readers want more detailed data, please feel free to contact the corresponding author (H.-C.W.).

## Supplementary Materials

The following supporting information can be downloaded at: https://www.mdpi.com/article/10.3390/diagnostics15131686/s1, Figure S1: Confusion matrices obtained by training and testing the YoloV3 model, where Figure S1a is the confusion matrix obtained by running the YoloV3 model on the WLI dataset, while Figure S1b is the confusion matrix obtained by running the YoloV3 model on the SAVE dataset; Figure S2: Training results in the form of plots for the YOLOv3 model, where Figure S2a shows the training results obtained by running the YoloV3 model on the WLI dataset, while Figure S2b displays the training results obtained by running the YoloV3 model on the SAVE dataset; Figure S3: Training results in the form of plots for the ScaledYOLOv4 model, where Figure S3a shows the training results obtained by running the ScaledYOLOv4 model on the WLI dataset, while Figure S3b displays the training results obtained by running the ScaledYOLOv4 model on the SAVE dataset; Figure S4: Confusion matrices obtained by training and testing the YOLOv6 model, where Figure S4a is the confusion matrix obtained by running the YOLOv6 model on the WLI dataset, while Figure S4b is the confusion matrix obtained by running the YOLOv6 model on the SAVE dataset; Figure S5: Precision curves obtained by training and testing the YOLOv6 model, where Figure S5a is the precision curve obtained by running the YOLOv6 model on the WLI dataset, while Figure S5b is the precision curve obtained by running the YOLOv6 model on the SAVE dataset; Figure S6: Recall curves obtained by training and testing the YOLOv6 model, where Figure S6a is the recall curve obtained by running the YOLOv6 model on the WLI dataset, while Figure S6b is the recall curve obtained by running the YOLOv6 model on the SAVE dataset; Figure S7: F1 curves obtained by training and testing the YOLOv6 model, where Figure S7a is the F1 curve obtained by running the YOLOv6 model on the WLI dataset, while Figure S7b is the F1 curve obtained by running the YOLOv6 model on the SAVE dataset; Figure S8: Precision–recall curves obtained by training and testing the YOLOv6 model, where Figure S8a is the precision-recall curve obtained by running the YOLOv6 model on the WLI dataset, while Figure S8b is the precision–recall curve obtained by running the YOLOv6 model on the SAVE dataset; Figure S9: Training results in the form of plots for the YOLOv7 model, where Figure S9a shows the training results obtained by running the YOLOv7 model on the WLI dataset, while Figure S9b displays the training results obtained by running the YOLOv7 model on the SAVE dataset; Figure S10: Confusion matrices obtained by training and testing the YOLOv7 model, where Figure S10a is the confusion matrix obtained by running the YOLOv7 model on the WLI dataset, while Figure S10b is the confusion matrix obtained by running the YOLOv7 model on the SAVE dataset; Figure S11: Precision curves obtained by training and testing the YOLOv7 model, where Figure S11a is the precision curve obtained by running the YOLOv7 model on the WLI dataset, while Figure S11b is the precision curve obtained by running the YOLOv7 model on the SAVE dataset; Figure S12: Recall curves obtained by training and testing the YOLOv7 model, where Figure S12a is the recall curve obtained by running the YOLOv7 model on the WLI dataset, while Figure S12b is the recall curve obtained by running the YOLOv7 model on the SAVE dataset; Figure S13: F1 curves obtained by training and testing the YOLOv7 model, where Figure S13a is the F1 curve obtained by running the YOLOv7 model on the WLI dataset, while Figure S13b is the F1 curve obtained by running the YOLOv7 model on the SAVE dataset; Figure S14: Precision–recall curves obtained by training and testing the YOLOv7 model, where Figure S14a is the precision–recall curve obtained by running the YOLOv7 model on the WLI dataset, while Figure S14b is the precision–recall curve obtained by running the YOLOv7 model on the SAVE dataset. Table S1. Dataset details before and after augmentation; Table S2. Average metric changes when switching from WLI to SAVE for each YOLO variant.
