# Supplementary material for: Optimizing Esophageal Cancer Diagnosis with Computer-Aided Detection by YOLO Models Combined with Hyperspectral Imaging
Source: Diagnostics (Basel). 2025 Jul 2;15(13):1686. doi: 10.3390/diagnostics15131686 (PMC12248576; doi:10.3390/diagnostics15131686)
Supplement: Supplementary file 1 [file diagnostics-15-01686-s001.zip › diagnostics-3669167-supplementary.pdf]

SUPLEMENTARY DOCUMENT

S1: DATSET

Table S1. Dataset details before and after augmentation

Class Name	No. of instances before augmentation	No. of instances after augmentation
0:Dysplasia	601	1202
1:SCC	159	318
2:Bleeding	213	426
3:Inflammation	81	162
4:through	1292	2584
5:Cardia	323	646
6:Object	643	1285
7:Tool	51	102
8:Artificial Information	164	328
9:Bubble	661	1322
10:Reflection	1068	2134
Total	2063	4125

Hyperparameter optimization proceeded in two stages using the fixed 20 % validation hold-out. First, we performed a coarse grid search over batch size {8, 16, 32} and initial learning rate {1e-4, 1e-3, 1e-2}. In the second stage, we applied Bayesian optimization (Tree-structured Parzen Estimator) to refine weight decay (1e-5–1e-3) and the IoU evaluation threshold (0.4–0.6). Anchor box priors were derived via k-means clustering on the training set, as implemented in YOLOv8. Optimal settings were found to be:

- Batch size: 32
- Initial learning rate: 1×10^{-3} with cosine decay to 1×10^{-6}
- Weight decay: 5×10^{-4}
- IoU threshold (NMS and evaluation): 0.5

All other parameters (momentum = 0.937, epochs = 300) were left at their default values. We confirmed stability by repeating training with three independent random splits—mean average precision varied by less than 0.5 %. Full k-fold cross-validation was not performed due to computational constraints, but will be adopted in future work to further validate robustness.

S2: Evaluation Indices

Precision:

Precision measures how well a model predicts the positive outcomes; it is also sometimes called Positive Predictive Value. It can be defined as the ratio of all positive forecasts to precisely predicted positive observations as shown in (s1)

$$\text{Precision} = \frac{\text{True Positives}}{\text{True Positives} + \text{False Positives}} \qquad (s1)$$

Recall:

Recall, also known as True Positive Rate or Sensitivity, measures how well a model can find each relevant event in a dataset. This represents the percentage of all observations made during the actual class that were all properly predicted to be positive as shown in (s2)

$$\text{Recall} = \frac{\text{True Positives}}{\text{True Positives} + \text{False Negatives}} \quad (s2)$$

F1 – Score:

The F1-Score is the harmonic mean of Precision and Recall. Because it provides a single metric that balances the trade-offs between precision and recall, it is useful for evaluating the overall performance of a classification model, especially when there is an imbalance between the classes as shown in (s3)

$$\text{F1} - \text{Score} = \frac{2 * \text{Precsion} * \text{Recall}}{\text{Precision} + \text{Recall}} \quad (s3)$$

mAP50:

Mean Average Precision (mAP) at 0.5 Intersection over Union is a metric used to evaluate object identification models (IoU). After calculating the average precision for each class in the dataset, it computes the mean of these average precisions. The IoU threshold of 0.5 determines whether a predicted bounding box is considered accurate if it overlaps the ground truth bounding box by at least 50% as shown in (s4)

$$mAP = \frac{1}{n}\sum_{k=1}^{k=n} AP_k \quad (s4)$$

Table S2. Average metric changes when switching from WLI to SAVE for each YOLO variant

Framework	ΔPrecision	ΔRecall	ΔF1	ΔmAP50
**ScaledYoloV4**	−3.12 %	+4.52 %	+1.75 %	+4.92 %
**YOLOV3**	+1.19 %	+2.67 %	+3.76 %	+2.50 %
**YOLOV5**	+2.48 %	−0.41 %	+1.16 %	−1.34 %
**YOLOV6**	+0.23 %	−1.93 %	−1.03 %	+1.19 %
**YOLOV7**	+2.04 %	+0.11 %	+1.08 %	+2.32 %
**YOLOV8**	+2.73 %	+5.47 %	+4.03 %	+14.10 %

Key observations:

- **YOLOv8** shows the largest and most consistent gains across all metrics, especially a substantial +14.1 % boost in mAP50, indicating greatly improved localization confidence with SAVE.
- **YOLOv7** and **YOLOv3** also benefit overall, with modest F1 improvements of +1.1 % and +3.8 %, respectively.
- **Scaled YOLOv4** sees reduced precision (−3.1 %) but gains in recall and mAP, suggesting SAVE helps it find more lesions at the cost of slightly more false positives.

- **YOLOv5** and **YOLOv6** show mixed results, with YOLOv5 losing some mAP (−1.34 %) and YOLOv6 dropping both recall and F1 slightly; however, both still exhibit positive precision gains.

S3: RESULTS

S3.1: YOLOv3

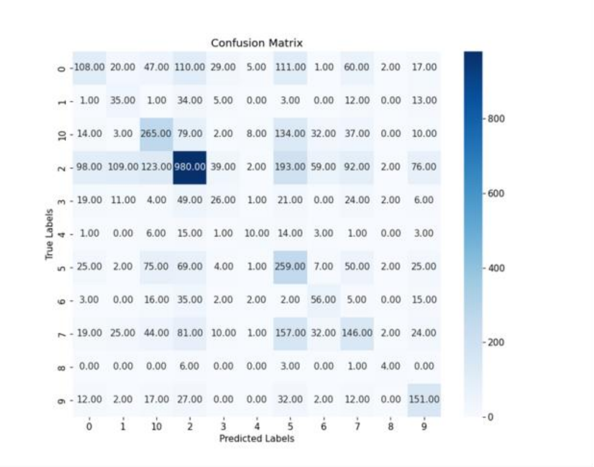

FigS1(a)

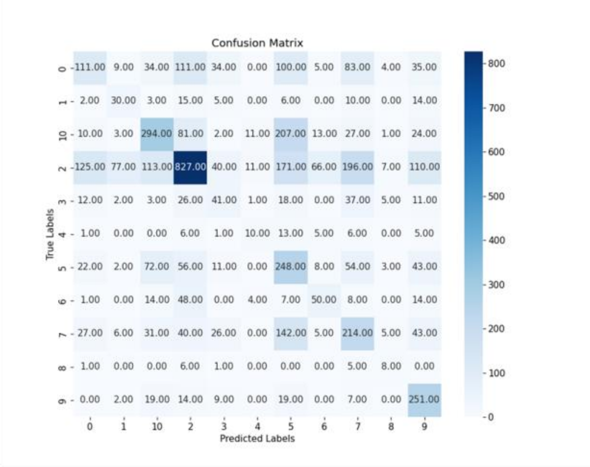

FigS1(b)

FigS1 contains the confusion matrices obtained by training and testing the YoloV3 model. Where FigS1(a) is the confusion matrix obtained by running the YoloV3 model on the WLI dataset, while FigS1(b) is the confusion matrix obtained by running the YoloV3 model on the SAVE dataset.

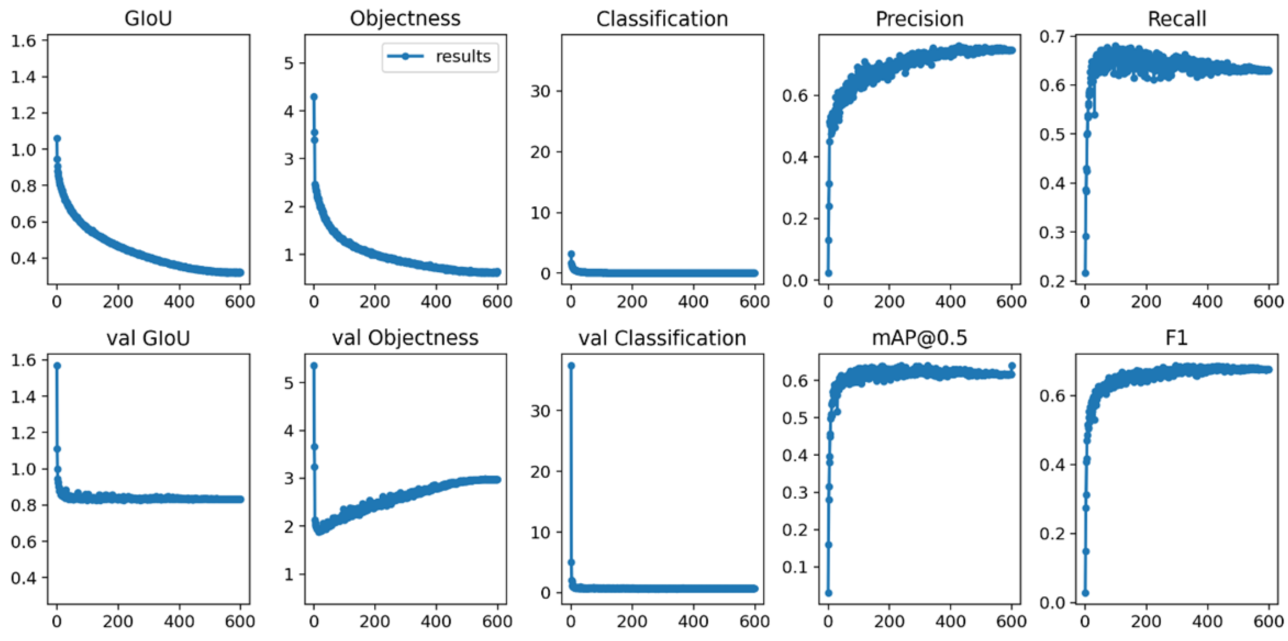

FigS2(a)

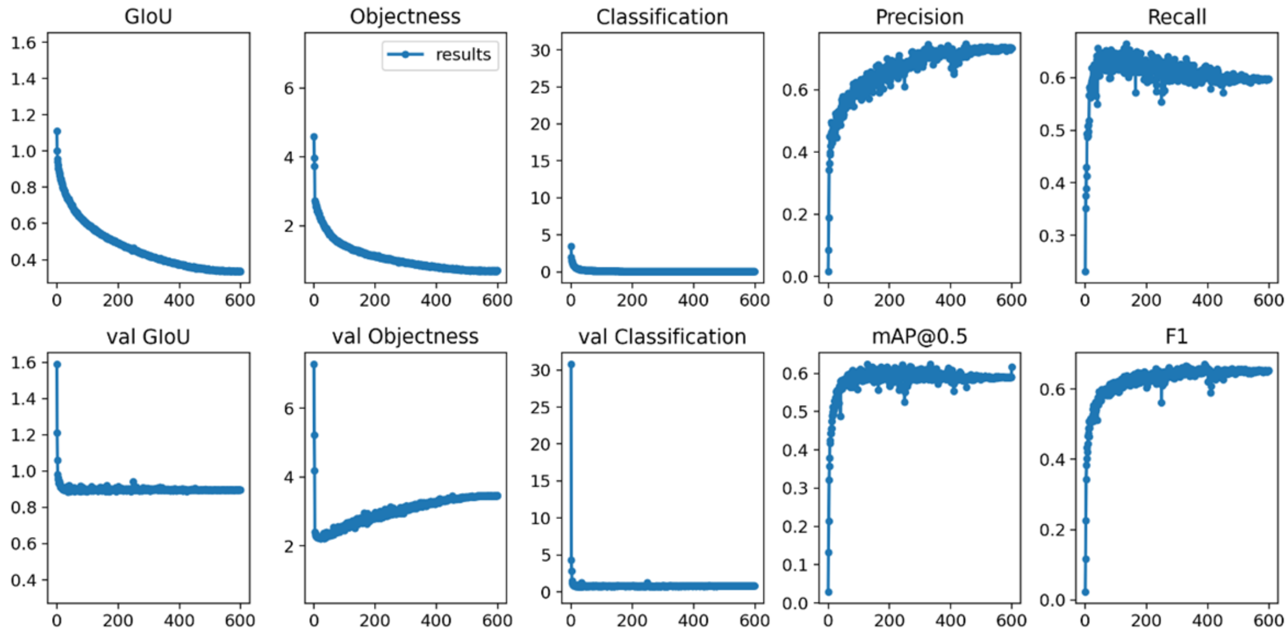

FigS2(b)

FigS2 contains training results in the form of plots for the YOLOv3 model. Where FigS2(a) is the training results plot obtained by running the YoloV3 model on the WLI dataset, while FigS2(b) is the training results plot obtained by running the YoloV3 model on the SAVE dataset.

S3.2: ScaledYOLOv4

S3.3: YOLOV6

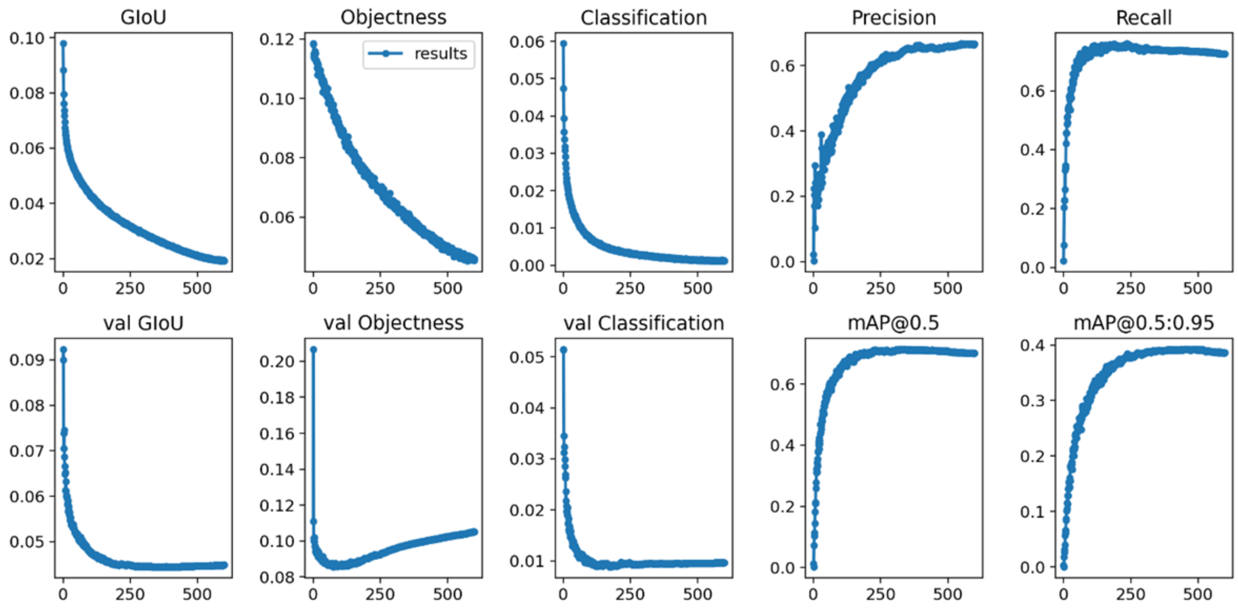

FigS3(a)

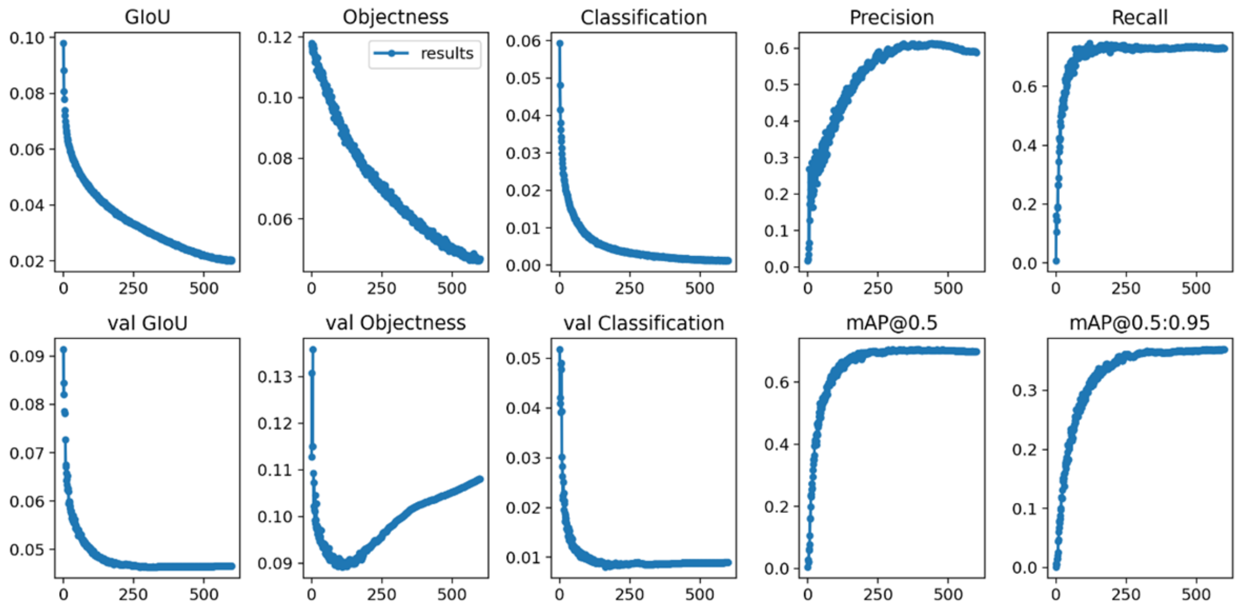

FigS3(b)

Fig S3. Contains training results in the form of plots for the ScaledYOLOv4 model. Where FigS3(a) is the training results plot obtained by running the ScaledYOLOv4 model on the WLI dataset, while FigS3(b) is the training results plot obtained by running the ScaledYOLOv4 model on the SAVE dataset.

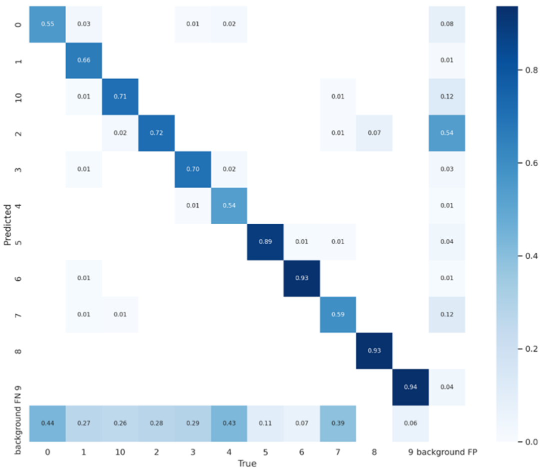

FigS4(a)

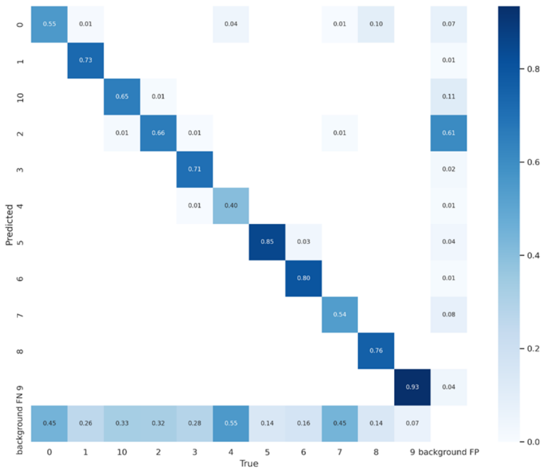

FigS4(b)

FigS4 contains the confusion matrices obtained by training and testing the YOLOv6 model. Where FigS4(a) is the confusion matrix obtained by running the YOLOv6 model on the WLI dataset, while FigS4(b) is the confusion matrix obtained by running the YOLOv6 model on the SAVE dataset.

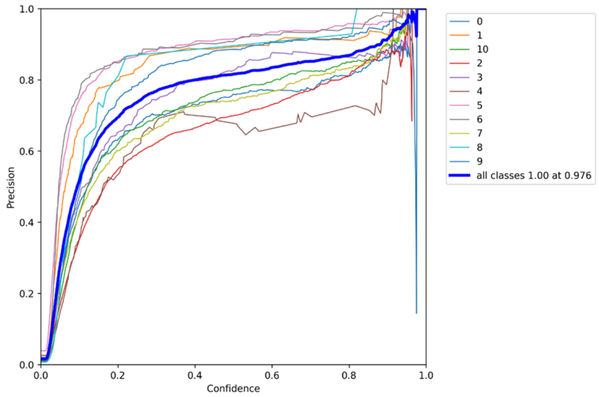

FigS5(a)

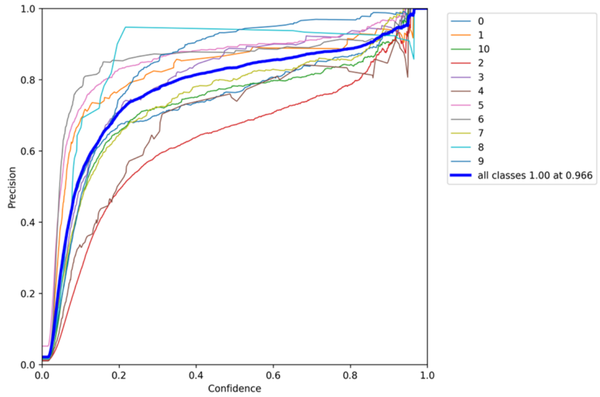

FigS5(b)

FigS5. Precision Curves for YOLOv6

FigS5 contains the precision curves obtained by training and testing the YOLOv6 model. Where FigS5(a) is the precision curve obtained by running the YOLOv6 model on the WLI dataset, while FigS5(b) is the precision curve obtained by running the YOLOv6 model on the SAVE dataset.

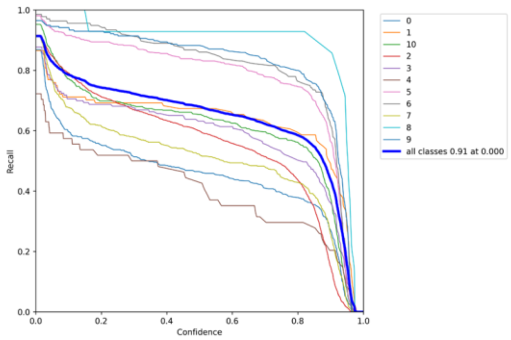

FigS6(a)

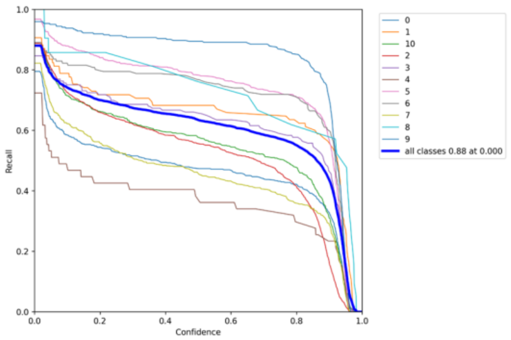

FigS6(b)

FigS6. Recall Curves for YOLOv6

FigS6 contains the recall curves obtained by training and testing the YOLOv6 model. Where FigS6(a) is the recall curve obtained by running the YOLOv6 model on the WLI dataset, while FigS6(b) is the recall curve obtained by running the YOLOv6 model on the SAVE dataset.

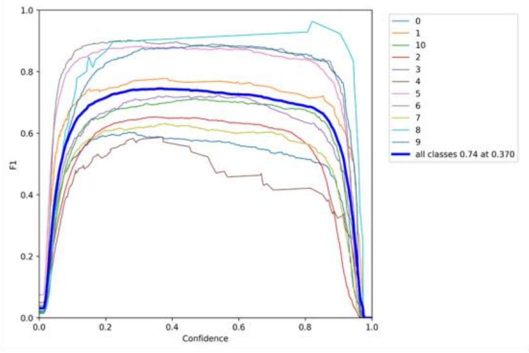

FigS7(a)

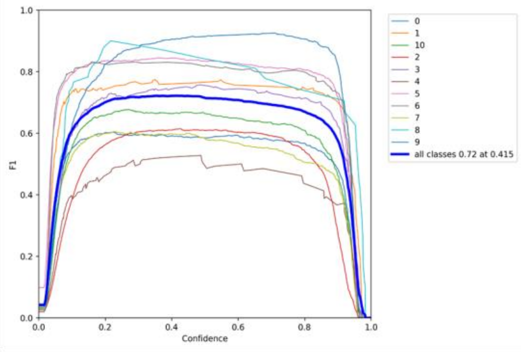

FigS7(b)

FigS7. F1-Score Curves for YOLOv6

FigS7 contains the F1 curves obtained by training and testing the YOLOv6 model. Where FigS7(a) is the F1 curve obtained by running the YOLOv6 model on the WLI dataset, while FigS7(b) is the F1 curve obtained by running the YOLOv6 model on the SAVE dataset.

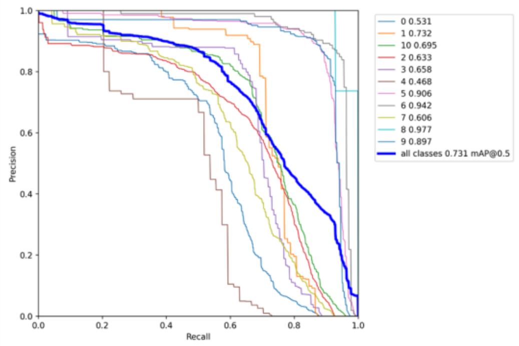

FigS8(a)

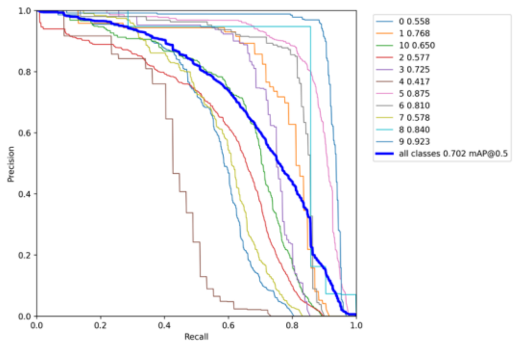

FigS8(b)

FigS8. Precision-Recall Curves for YOLOv6

FigS8 contains the precision-recall curves obtained by training and testing the YOLOv6 model. Where FigS8(a) is the precision-recall curve obtained by running the YOLOv6 model on the WLI dataset, while FigS8(b) is the precision-recall curve obtained by running the YOLOv6 model on the SAVE dataset.

S3.4: YOLOv7

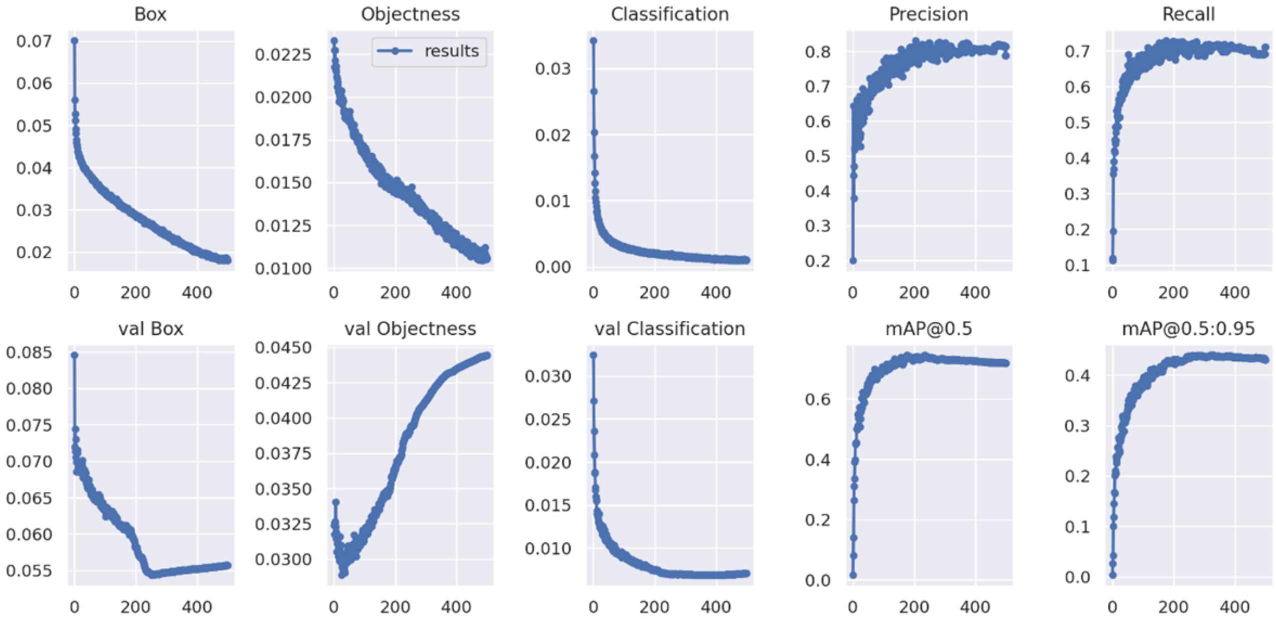

FigS9(a)

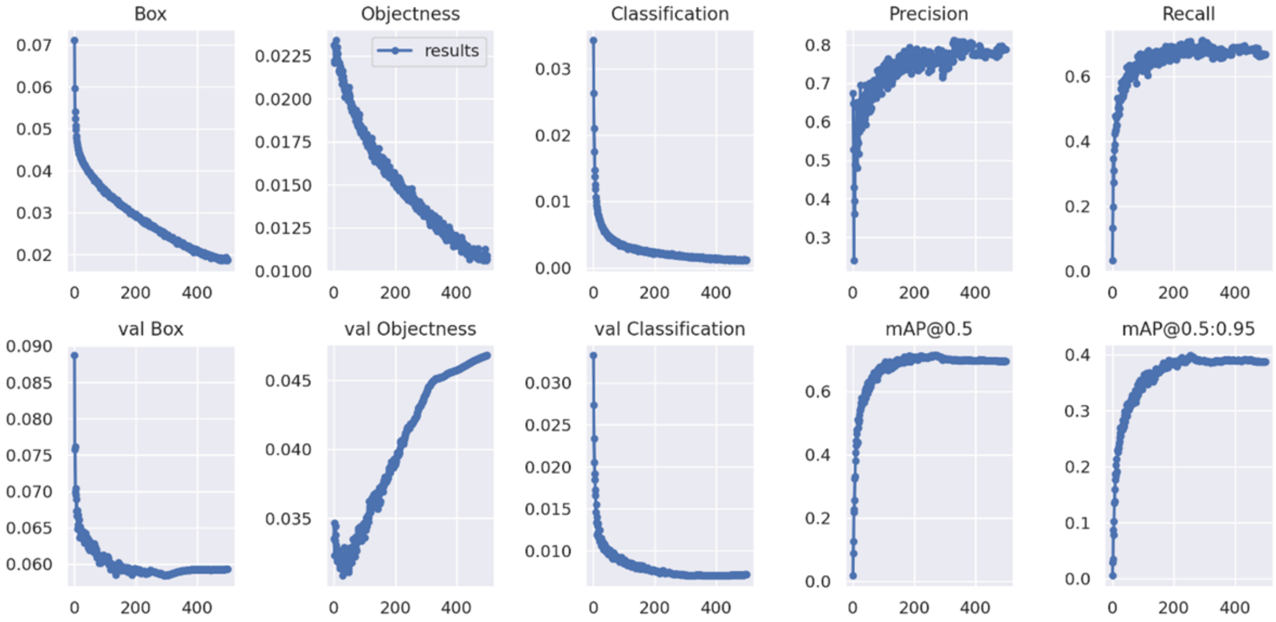

FigS9(b)

FigS9. Training Results for YOLOv7

Fig S9 contains training results in the form of plots for the YOLOv7 model. Where FigS9(a) is the training results plot obtained by running the YOLOv7 model on the WLI dataset, while FigS9(b) is the training results plot obtained by running the YOLOv7 model on the SAVE dataset.

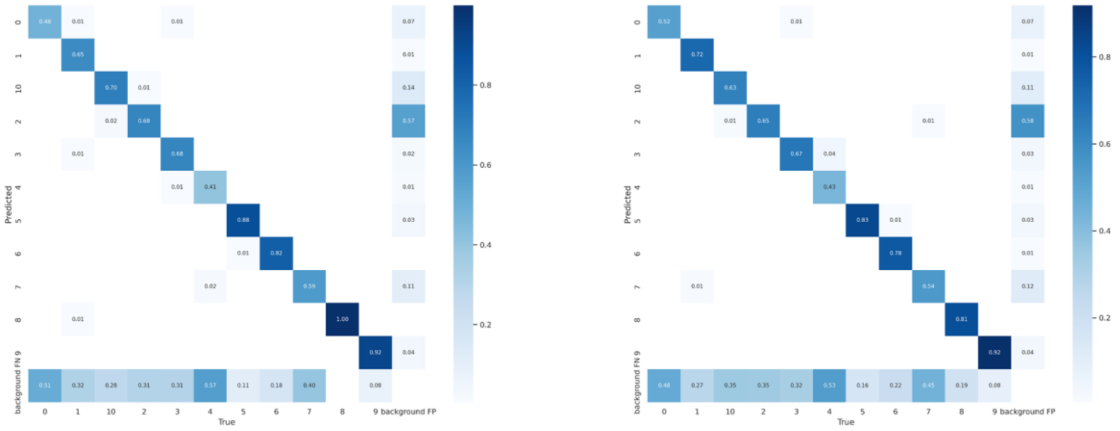

FigS10(a) FigS10(b)

FigS10. Confusion Matrices for YOLOv7

FigS10 contains the confusion matrices obtained by training and testing the YOLOv7 model. Where FigS10(a) is the confusion matrix obtained by running the YOLOv7 model on the WLI dataset, while FigS10(b) is the confusion matrix obtained by running the YOLOv7 model on the SAVE dataset.

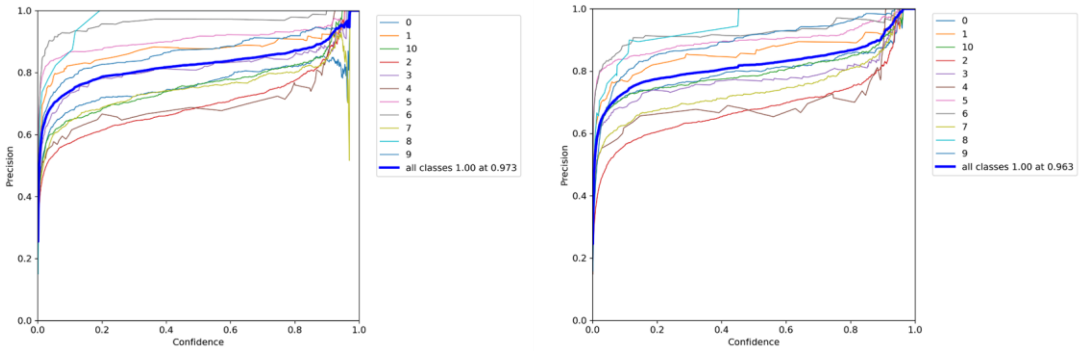

FigS11(a) FigS11(b)

FigS11. Precision Curves for YOLOv7

FigS11 contains the precision curves obtained by training and testing the YOLOv7 model. Where FigS11(a) is the precision curve obtained by running the YOLOv7 model on the WLI dataset, while FigS11(b) is the precision curve obtained by running the YOLOv7 model on the SAVE dataset.

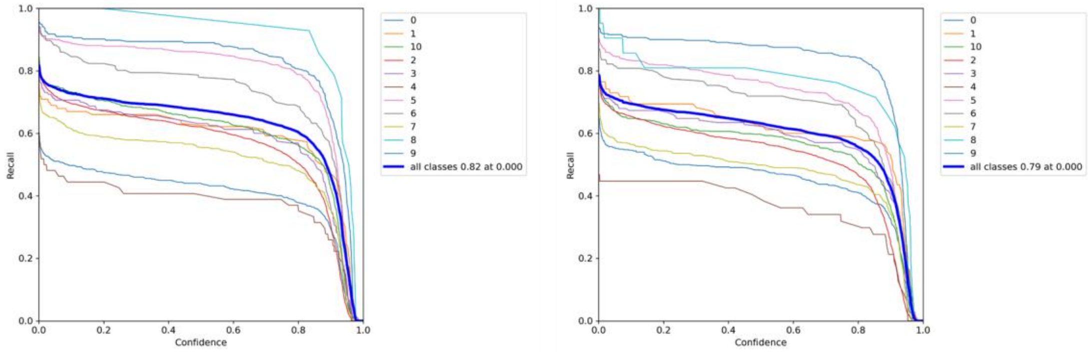

FigS12(a) FigS12(b)

FigS12. Recall Curves for YOLOv7

FigS12 contains the recall curves obtained by training and testing the YOLOv7 model. Where

FigS12(a) is the recall curve obtained by running the YOLOv7 model on the WLI dataset, while FigS12(b) is the recall curve obtained by running the YOLOv7 model on the SAVE dataset.

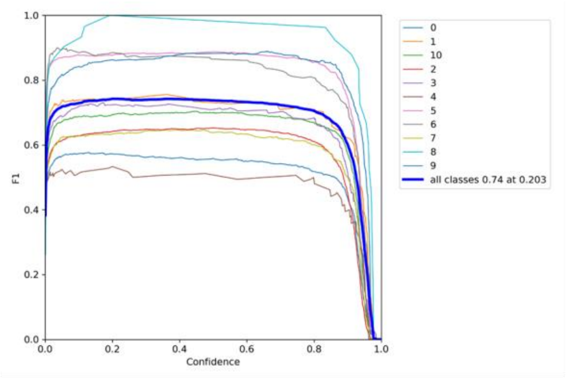

FigS13(a)

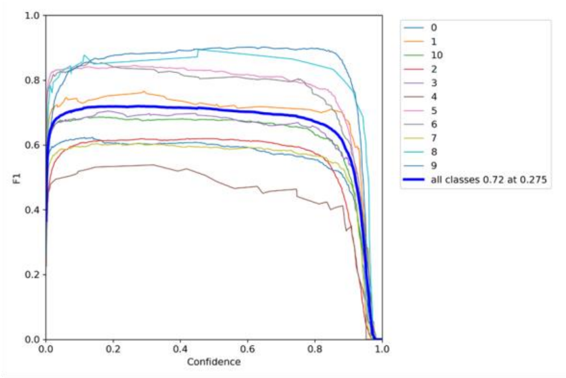

FigS13(b)

FigS13. F1-Score Curves for YOLOv7

FigS13 contains the F1 curves obtained by training and testing the YOLOv7 model. Where FigS13(a) is the F1 curve obtained by running the YOLOv7 model on the WLI dataset, while FigS13(b) is the F1 curve obtained by running the YOLOv7 model on the SAVE dataset.

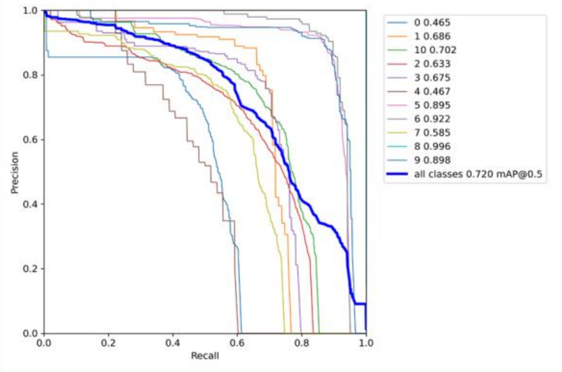

FigS14(a)

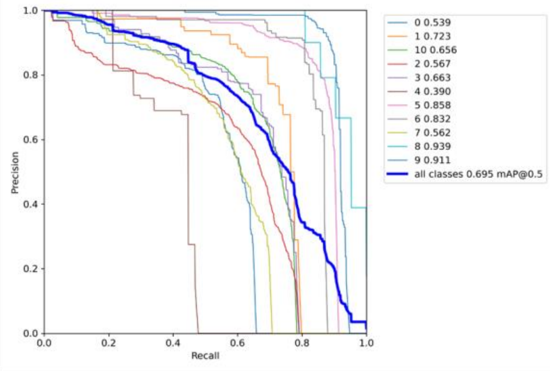

FigS14(b)

FigS14. Precision-Recall Curves for YOLOv7

FigS14 contains the precision-recall curves obtained by training and testing the YOLOv7 model. Where FigS14(a) is the precision-recall curve obtained by running the YOLOv7 model on the WLI dataset, while FigS14(b) is the precision-recall curve obtained by running the YOLOv7 model on the SAVE dataset.
